# Device-Free Wireless Sensing for Gesture Recognition Based on Complementary CSI Amplitude and Phase

**DOI:** 10.3390/s24113414

**Published:** 2024-05-25

**Authors:** Zhijia Cai, Zehao Li, Zikai Chen, Hongyang Zhuo, Lei Zheng, Xianda Wu, Yong Liu

**Affiliations:** 1 School of Electronics and Communication Engineering, Guangzhou University, Guangzhou 510006, China; 2 School of Information and Optoelectronic Science and Engineering, South China Normal University, Guangzhou 510006, China; 3 School of Physics and Telecommunication Engineering, South China Normal University, Guangzhou 510006, China; 4 School of Electronics and Information Engineering, South China Normal University, Foshan 528225, Chinayliu@m.scnu.edu.cn (Y.L.)

**Keywords:** human gesture recognition, WiFi-based wireless sensing, channel state information

## Abstract

By integrating sensing capability into wireless communication, wireless sensing technology has become a promising contactless and non-line-of-sight sensing paradigm to explore the dynamic characteristics of channel state information (CSI) for recognizing human behaviors. In this paper, we develop an effective device-free human gesture recognition (HGR) system based on WiFi wireless sensing technology in which the complementary CSI amplitude and phase of communication link are jointly exploited. To improve the quality of collected CSI, a linear transform-based data processing method is first used to eliminate the phase offset and noise and to reduce the impact of multi-path effects. Then, six different time and frequency domain features are chosen for both amplitude and phase, including the mean, variance, root mean square, interquartile range, energy entropy and power spectral entropy, and a feature selection algorithm to remove irrelevant and redundant features is proposed based on filtering and principal component analysis methods, resulting in the construction of a feature subspace to distinguish different gestures. On this basis, a support vector machine-based stacking algorithm is proposed for gesture classification based on the selected and complementary amplitude and phase features. Lastly, we conduct experiments under a practical scenario with one transmitter and receiver. The results demonstrate that the average accuracy of the proposed HGR system is 98.3% and that the F1-score is over 97%.

## 1. Introduction

With the ongoing development of information and communication technology, human–computer interaction (HCI) has become an emerging paradigm, within which human gesture recognition (HGR) plays a significant role [1,2]. Thus, how to develop an efficient and robust HGR system that can recognize the gestures captured by diverse devices and map them to the specific commands is a critical issue. Because human behaviors can greatly affect the wireless propagation environment, wireless sensing technology based on WiFi, which captures the dynamic characteristics of WiFi channels to analyze the human behaviors, is a promising paradigm. It integrates sensing capability into wireless communication, and shows potential to realize contactless and non-line-of-sight (NLoS) sensing while protecting privacy. In this paper, we aim to design an efficient device-free gesture recognition system based on wireless sensing technology.

For HGR, most existing research works can be categorised into methods based on visual, sensor, or wireless sensing. By deploying cameras to capture gestures, vision-based HGR systems, i.e., Leap Motion [3] and Kinect [4], can achieve a high accuracy in detecting human behaviors. However, the line-of-sight (LoS) requirement and the privacy leakage issue restrict the application scenarios of vision-based methods. In sensor-based methods, i.e., Wiimote [5], dedicated sensors are equipped to capture hand movements. However, this can be inconvenient due to the requirement of human cooperation, and energy supply remains a great challenge for sensor based methods. Among wireless sensing based methods, mmWave is characterized by its high frequency and strong anti-jamming ability [6,7], allowing it to be applied for human gesture recognition. However, high signal attenuation and the requirement for specialized hardware units limit its application for human gesture recognition. Recently, WiFi-based wireless sensing technology has attracted much research attention due to its advantages of passive and NLoS sensing capability and independence from the lighting environment [8,9,10].

An HGR system called WiGest was proposed in [11], which explored the fluctuating characteristics of the received signal strength indicator (RSSI) to recognize seven gestures and then control a media player application. Compared with RSSI, the channel state information (CSI) can provide more useful information to unveil the impact of human gestures on wireless channels. CSI amplitude is exploited in the WiGeR system proposed in [12], where an effective segmentation algorithm based on wavelet analysis and short-time energy was also proposed. Similarly, WriFi, proposed in [13], uses the energy of the CSI amplitude based on the fast Fourier transform (FFT) to continuously detect air writing gestures. In addition to the CSI amplitude, the fine-grained CSI phase can be used for HGR system. In [14], the time and frequency features of the CSI phase were explored in the proposed PWiG system. WiADG, proposed in [15], constructs CSI frames as the input data of the classifier based on the phase difference among the receiving antennas, then uses a convolutional neural network (CNN)-based classifier to extract the most discriminative features from the CSI frames for to mapping to a domain-invariant latent feature space. In [16], a novel approach for converting CSI data into images and inputting them to a 2D-CNN was proposed, resulting in improved recognition accuracy. The authors validated that attention-based bidirectional long short term memory (BiLSTM) could be further exploited to improve system performance. In [17], an attention mechanism-based BiLSTM model was proposed for passive human activity recognition using CSI signals. In this approach, representative features are learned through BiLSTM, after which different weights are assigned to the learned features through the attentional mechanism. Experiments validated the effectiveness of this model. These works exploit either the CSI amplitude or phase to realize the gesture recognition. Thus, it seems natural to utilize the amplitude and phase jointly to improve the recognition accuracy. To achieve this, an HGR system called WiGrus was proposed in [18]. In this approach, the phase and amplitude features are extracted based on two feature extraction mechanisms, then combined to form a feature space. In addition, a two-stage random forest algorithm was proposed for gesture recognition. Meanwhile, an embedded approach can be used to address the sensitivity of the CSI over multiple subcarriers. The accuracy can be guaranteed only if the feature selection classifier is also used for new data samples. In fact, the CSI amplitude and phase are complementary to each other [19], which means that their sensitivities to human gestures can mutually cancel. To the best of our knowledge, this complementary characteristic has not yet been fully exploited for HGR systems, especially concerning diverse sensitivities of CSI over multiple subcarriers, which motivates our present work.

In this paper, we study a WiFi sensing-based human gesture recognition system consisting of modules for data collection, data processing, feature extraction and selection, and training and gesture recognition. To improve the quality of the original CSI amplitude and phase, operations for eliminating the phase deviation based on a linear transform and removing abnormal data and background noise are successively conducted in the data processing module. Then, in the feature extraction and selection module, six time and frequency domain features which can effectively describe the CSI in terms of the sample data distribution, frequency band distribution, and spectral structure are chosen, namely, the mean, variance, root mean square (RMS), interquartile range (IQR), energy entropy (EE) [20], and power spectral entropy (PSE) [21]. This greatly reduces the system’s computation time while preserving the original sample’s separability. To select the feature subspaces from a large number of features, the filtering method, which is widely adopted in feature selection [22], is utilized to remove irrelevant features. Meanwhile, the feature reduction method is used to remove redundant features. Feature selection and reduction can effectively reduce the complexity of the algorithm while guaranteeing its performance. Furthermore, we propose a classification fusion estimation scheme based on a stacking algorithm that jointly exploits the complementary CSI amplitude and phase. Our experimental results validate the performance of the proposed HGR system. In summary, the main contributions of this paper are as follows:

- A WiFi-based gesture recognition system in which the complementary CSI amplitude and phase are jointly exploited for the classification fusion estimation.
- An improved Fisher method for feature selection which incorporates the distances among category centers to calculate the Fisher score, effectively avoiding misunderstandings caused by the overlap between different categories.
- A classification fusion estimation approach based on a stacking algorithm which in which the CSI amplitude and phase models are trained separately, then a meta-model is constructed to exploit the complementary characteristics of the phase and amplitude.
- Our experiments show that the average accuracy of the proposed HGR system is 98.3%.

The rest of this paper is organized as follows: Section 2 reviews related research works on wireless sensing and gesture recognition, especially WiFi sensing based gesture recognition; Section 3 presents the details of the proposed device-free gesture recognition system, including overview framework, data collection, data processing, feature extraction and selection, and training and gesture recognition; Section 4 presents the experimental evaluation; finally, Section 5 discusses limitations and challenges and concludes the paper.

## 2. Related Works

In this section, we review the extensive existing research works on WiFi-based wireless sensing and gesture recognition, especially WiFi-based gesture recognition technology.

### 2.1. WiFi-Based Wireless Sensing

In 2000, Bahl et al., firstly exploited the received signal strength (RSS) of WiFi signals to design an indoor positioning system [23]. This was the pioneering work utilizing WiFi signals for wireless sensing operation. Subsequently, the use of WiFi signals for human behavior recognition has attracted much research attention, including the detection/recognition/localization of indoor persons [24,25,26,27,28,29], identification of human gestures [10,18] and human falls/activities/language [9,30,31], and more.

In [32], NLoS sensing of human actions was achieved by collecting WiFi signals and analyzing the Doppler frequency shifts (DFS). Zhou et al. exploited RSSI to detect the presence of indoor people in [29], in which their proposed scheme first introduced the concept of omnidirectional passive human detection. In [33], a CSI tool was released to greatly facilitate the acquisition of fine-grained CSI data from commercial WiFi network interface cards (NICs), enabling more fine-grained identification than with coarse-grained RSS and RSSI. To recognize human gestures, a gesture recognition system called Wikey was proposed in [34] which recognized 37 keys on a computer keyboard. A language detection system called WiHear was proposed in [30]. The authors showed that WiFi signals could determine what a person was saying by building a verbal model for each syllable. In [31], a human fall detection system called WiFall was developed, exploring finer-grained CSI to aid in health monitoring of elderly persons. The complementary characteristic of the CSI amplitude and phase was demonstrated in [19], where it was utilized to eliminate “blind spots”. In general, WiFi-based wireless sensing technology has become a promising paradigm for human behavior sensing and recognition. It has been deployed for various applications, including NLoS sensing, omnidirectional passive detection, fine-grained signal acquisition and recognition, and and exploration of various human behavior recognition systems.

### 2.2. Gesture Recognition

Gestures can come from actions of all parts of a person’s body, but generally refer to actions of the face and hands. Humans can use simple gestures to control or interact with devices, enabling computers to understand human behavior. Generally speaking, gesture recognition schemes can be divided into three categories: wearable sensor-based methods [35,36], vision-based methods [37,38] and WiFi-based methods [39,40,41].

#### 2.2.1. Wearable Sensor-Based Methods

Wearable sensor-based gesture recognition techniques are mainly used to achieve gesture recognition by attaching sensors (such as acceleration sensors [42]) to the human body and collecting relevant motion information [43]. Despite the low cost of acceleration sensors and their easy large-scale application, sensors embedded in sensory gloves which directly adhere to the skin of the hand have gained the most attention thanks to their ability to collect more accurate hand movement data. Sensory gloves equipped with bending sensors and inertial sensors can be worn to sense hand movements and enable gesture recognition, as in [44,45]. Compared to acceleration sensors and sensory gloves, electromyography (EMG) signals can reflect subtle changes in human muscle movement, enabling the recognition of more refined and complex gestures, as in [46,47]. More interestingly, activity recognition can be achieved through sensors integrated into smartphones. In [48], acceleration, gyroscope, and magnetometer data from a cyclist’s smartphone were obtained through a sensor logger, then a 1D-CNN-BiLSTM model based on an attention mechanism was used to detect the cyclist’s activity. Although the sensor-based gesture recognition approach can accurately recognize movements, it requires users to wear special sensors, resulting in poor user comfort.

#### 2.2.2. Vision-Based Methods

Early work has provided a solid foundation for gesture recognition using dedicated cameras [49,50]. Computer vision-based gesture recognition technology usually involves cameras deployed to acquire images or videos containing gesture information. After processing the resulting images via denoising and segmentation, the features of the gestures in the image are used for training. Gesture recognition in images or videos can be realized by techniques such as pattern recognition, as in [51,52]. Although vision based gesture recognition does not require the user to wear any sensors, it has specific brightness requirements and cannot work in the dark. In addition, vision-based gesture recognition only works in LOS scenarios, and invades the user’s privacy.

#### 2.2.3. WiFi-Based Wireless Sensing Methods

Compared with the above two methods, commonplace WiFi technology has been exploited for wireless sensing due to its ubiquity, low cost, and easy scalability. The CSI of communication links under specific scenarios can be acquired by widely deployed WiFi devices. The CSI features can then be further extracted and used for training to unveil the effects of diverse human gestures on WiFi channels for full human gesture recognition. For example, WiGAN [53] removes noise and extracts amplitude features, then uses a generative adversarial network to generate new signals that resemble the training data for each gesture. The discriminator compares the generated signals with the real signals to determine specific gestures. In [54], a novel motion pause buffer was proposed to address issues caused by short gesture pauses. The CSI is captured for different gestures, then the gesture segments are extracted by detecting the change of the preprocessed CSI amplitude. Subsequently, the CSIs of multiple antenna pairs were combined to construct a gesture image, allowing the gesture recognition problem to be transformed into an image classification problem. A fine-tuned CNN model was then used to extract the high-level features from the gesture images, which were in turn exploited to recognize the gestures. WiHGR [55] adopts a sparse recovery method to identify the primary propagation path of WiFi channels, based on which it constructs a phase difference matrix by computing the phase difference between the primary propagation paths of adjacent receiving antennas. Subsequently, an improved attention based bidirectional gated recurrent unit (BGRU) network is exploited to extract features and perform training from the phase difference matrix. A lightweight few-shot learning network called WiGR was proposed in [56] to address the problem of hard domain shift, consisting of a feature extraction subnetwork and a similarity discrimination subnetwork. The learning network introduces lightweight and efficient blocks to reduce computational complexity and achieve high performance. The feature extraction subnetwork uses a 2D convolutional kernel to simultaneously extract the spatial features and temporal dynamics of the CSI phase filtered by finite impulse response. The similarity discrimination subnetwork utilizes a learning-based neural network as the similarity measurement method to determine the gestures. Furthermore, the authors created a CSI-domain adaptation dataset containing CSI traces with various domain factors to simulate real-world scenarios. In [57], a method based on higher-order statistics was proposed to extract third-order cumulant features from original CSI data. In this method, feature extraction is performed directly from the CSI by introducing third-order cumulant estimation, with a multi-level Support Vector Machine (SVM) classifier with radial basis function (RBF) kernel theb used for gesture recognition. A robust device-free number gesture recognition approach was proposed in [58]. First, phase calibration and preprocessing were performed on the collected CSI. Second, the amplitude difference is used to detect gesture segments, as the CSI amplitude difference provides a better basis signal than the phase difference for recognizing gesture transition endpoints. After normalizing the amplitude and phase of the three antennas, their average is used as the input for feature extraction, with a four-layer deep learning model deployed to extract features. Finally, the features are used as input to the classification model to achieve device-free number gesture recognition. Wi-SL [59] is a gesture recognition method based on the CSI amplitude and phase difference in which the normalized amplitude and phase difference covariance matrices are extracted and then combined as features. In [60], a novel classification model was proposed to recognize driver gestures for vehicle infotainment system applications by exploiting sparse representation-based classification and a variant of the k-nearest neighbors algorithm. First, the proposed method linearly calibrates the measured CSI phase, filters the measured CSI amplitude, then detects gesture segments based on the variance of amplitude and phase. Second, PCA is used to extract the second, third, and fourth principal components from the subcarriers. Finally, six statistical features are extracted from the principal components, then six phase features and six amplitude features are combined into a feature vector, which forms the input data used by the classification model to realize driver gesture recognition. In summary, the gesture recognition approach based on WiFi wireless sensing shows great potential in terms of NLoS and contactless sensing capability. The CSI of WiFi communication links consists of fine-grained information, and is being increasingly exploited by gesture recognition techniques thanks to its more sensitive to environmental changes; however, a lack of sample feature space selection and the complementary characteristics of the CSI amplitude and phase restrict the recognition performance. In this paper, we aim to develop a device-free gesture recognition system that can exploit the complementary characteristics of the CSI amplitude and phase, along with a corresponding classification fusion estimation algorithm.

## 3. Human Gesture Recognition System Based on Wireless Sensing

### 3.1. Framework Overview

**Figure 1:**
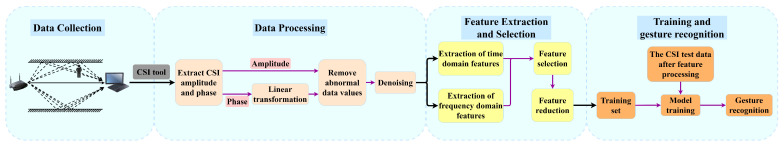
The proposed HGR system framework.

In this paper, we develop a device-free HGR system based on WiFi sensing technology, as illustrated in Figure 1. The HGR system consists of four modules: data collection, data processing, feature extraction/selection, and gesture trainning/recognition. In the data collection module, dedicated sensing devices are not necessary, and the universal WiFi transceiver is explored to measure and collect the CSI (including complementary amplitude and phase) under different gestures scenarios. Then, to improve the quality of CSI data, some processing operations are conducted, i.e., deleting abnormal data and denoising On this basis, six features of CSI data in the time and frequency domains are taken into account and a feature selection algorithm is investigated. Finally, a classification algorithm is proposed to train and recognize human gestures. All symbols and notations used in this paper are listed in Table 1.

### 3.2. Data Collection

The external environment greatly affects the channel conditions of communication links. Wireless sensing technology can be used to reveal and distinguish the factors that cause the dynamic characteristic of wireless channel, by collecting and analyzing the CSI data under different scenarios, e.g., different gestures.

The hardware unit of data collection consists of a WiFi access point (AP) and a mobile terminal with multiple antennas. When the AP is transmitting its information, the terminal is able to receive the radio frequency (RF) signals, then estimate and collect the corresponding CSI. In general, orthogonal frequency division multiplexing (OFDM) is exploited in WiFi, which means that the actual transmission channel consists of multiple orthogonal subchannels with different frequency bands (i.e., subcarriers). Therefore, the CSI is the channel frequency response (CFR) between each pair of transmitting and receiving antennas over 30 OFDM subcarriers. For example, when the AP and mobile terminal are deployed with one and three antennas, respectively, the CSI can be expressed as a 3 × 30 matrix, i.e.,

(1) $$H\left(t\right)=\left[\begin{array}{cccc} H_{1,1}\left(t\right) & H_{1,2}\left(t\right) & \cdots & H_{1,30}\left(t\right)\\ H_{2,1}\left(t\right) & H_{2,2}\left(t\right) & \cdots & H_{2,30}\left(t\right)\\ H_{3,1}\left(t\right) & H_{3,2}\left(t\right) & \cdots & H_{3,30}\left(t\right) \end{array}\right],$$

where H(t) is the CSI at time *t* and $H_{i,f}\left(t\right)$ is the CSI of the *i*-th transceiver antenna pair in the *f*-th subcarrier at time *t*. Meanwhile, $H_{i,f}\left(t\right)$ is rewritten as

(2) $$H_{i,f}\left(t\right)=\frac{Y_{i,f}\left(t\right)}{X_{i,f}\left(t\right)}=A_{i,f}\left(t\right)e^{j\varphi_{i,f}\left(t\right)},$$

where $X_{i,f}\left(t\right)$ and $Y_{i,f}\left(t\right)$ denote the transmitted and received signals, respectively, while $A_{i,f}\left(t\right)$ and $\varphi_{i,f}\left(t\right)$ are the amplitude and phase of $H_{i,f}\left(t\right)$, respectively. Because the theoretical channel model of CSI has been extensively discussed in [61,62], we do not repeat it here.

### 3.3. Data Processing

Because the received signals are affected by the background noise and frequency shift, there are several important issues that need to be addressed for the collected CSI, e.g., phase deviation, abnormal data, and noise, before the original CSI can be used for the recognition procedure. To address this, a number of essential processing operations are performed. Accordingly, the collected CSI phase is first processed to eliminate phase deviations, then the CSI amplitude and phase are processed by deleting abnormal data and eliminating background noise.

#### 3.3.1. Eliminating Phase Deviation

Usually, deviation exists between the true CSI phase and the measured phase. To improve the recognition accuracy, it is necessary to eliminate this CSI phase deviation. As presented in [63], the measured CSI phase of the *i*-th transceiver antenna pair in the *f*-th subcarrier is provided by

(3) $${\dot{\varphi }}_{i,f}\left(t\right)=\varphi_{i,f}\left(t\right)-2\pi \frac{k_{f}}{N}\delta \left(t\right)+\beta \left(t\right)+Z\left(t\right),$$

where $\varphi_{i,f}\left(t\right)$ is the true phase, δ(t) is the time offset between the transmitter and receiver, β(t) is the unknown phase offset, Z(t) is the noise introduced by the measurement procedure, $k_{f}$ is the subcarrier index varying from −28 to 28 in IEEE 802.11n, and *N* is the number of (FFT) window points. Essentially, the components δ(t) and β(t) can be eliminated via linear transformation. To realize this, we define two variables $a_{i}\left(t\right)$ and $b_{i}\left(t\right)$ as follows:

(4) $$a_{i}\left(t\right)=\frac{{\dot{\varphi }}_{i,n}\left(t\right)-{\dot{\varphi }}_{i,1}\left(t\right)}{k_{n}-k_{1}}=\frac{\varphi_{i,n}\left(t\right)-\varphi_{i,1}\left(t\right)}{k_{n}-k_{1}}-\frac{2\pi }{N}\delta \left(t\right),$$

(5) $$b_{i}\left(t\right)\;=\;\frac{1}{n}\;\sum_{f=1}^{n}{\dot{\varphi }}_{i,f}\left(t\right)\;=\;\frac{1}{n}\;\;\sum_{f=1}^{n}\;\varphi_{i,f}\left(t\right)\;-\;\frac{2\pi \delta (t)}{nN}\sum_{f=1}^{n}k_{f}\;+\;\beta \left(t\right),$$

where *n* is the total number of subcarriers. According to the IEEE 802.11n protocol, the frequencies of the subcarriers are perfectly symmetric, that is, $\sum_{f=1}^{n}k_{f}=0$. Therefore, $b_{i}\left(t\right)$ can be rewritten as

(6) $$b_{i}\left(t\right)=\frac{1}{n}\sum_{f=1}^{n}\varphi_{i,f}\left(t\right)+\beta \left(t\right).$$

Then, the linear expression $a_{i}\left(t\right)k_{f}+b_{i}\left(t\right)$ can be subtracted from the measured phase ${\dot{\varphi }}_{i,f}\left(t\right)$ to obtain the calibrated phase information of the *f*-th subcarrier, ${\tilde{\varphi }}_{i,f}\left(t\right)$, as follows:

(7) $$\begin{array}{cc} {\tilde{\varphi }}_{i,f}\left(t\right) & ={\dot{\varphi }}_{i,f}\left(t\right)-a_{i}\left(t\right)k_{f}-b_{i}\left(t\right)\\ & =\varphi_{i,f}\left(t\right)\;-\;\frac{\varphi_{i,n}\left(t\right)\;-\;\varphi_{i,1}\left(t\right)}{k_{n}-k_{1}}k_{f}\;-\;\frac{1}{n}\;\sum_{f=1}^{n}\varphi_{i,f}\left(t\right). \end{array}$$

To this end, the linear components δ(t) and β(t) are eliminated when the measurement noise Z(t) is small.

**Figure 2:**
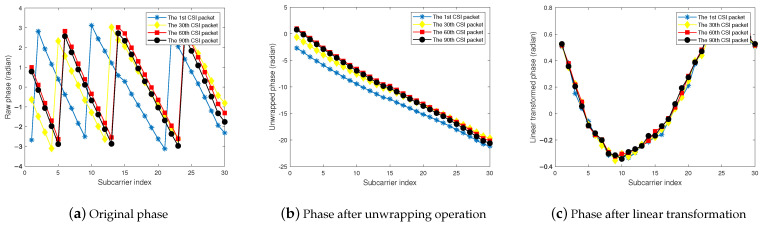
CSI phases of some subcarriers.

In Figure 2, we take an example of CSI phase from practical experiment to validate the effect of the phase deviation elimination procedure, where the phases of all subcarriers for the first, thirtieth, sixtieth, and ninetieth CSI samples of the second transceiver antenna pair are presented. The original CSI phases are shown in Figure 2a. It can be seen that the phases of the different subcarriers vary from −π to π, and are inverted at the critical points π and −π. Therefore, it is necessary to perform the phase unwrapping procedure on the original CSI phases. When the absolute value of the difference between two CSI phases before and after detection exceeds π, there is considered to be an inversion of the phase; at this point, the CSI phase is increased or decreased by 2π to ensure that the absolute value of the difference between the two CSI phases before and after does not exceed π, and so on for all of the phase values obtained from the measurement. After the phase unwrapping process, the original phase can be expanded into a continuous form in this way, as shown in Figure 2b. This process can be implemented using the unwrap function in MATLAB. Subsequently, based on the above theoretical analysis, the calibrated phase ${\tilde{\varphi }}_{i,f}\left(t\right)$ is obtained by eliminating the noise and phase deviation components via linear transformation, which is shown in Figure 2c.

#### 3.3.2. Replacing Abnormal Data

Among the collected CSI, there always exist some abnormal data which are much higher or lower than the mean and standard deviation of the CSI samples. In general, the 3σ criterion is an effective method for deleting and replacing such abnormal data, defined as values that deviate from the mean by more than three times the standard deviation. When such abnormal data are found, they are replaced with the mean value of the CSI samples.

If the time series window size is set to 7, then the mean $\mu_{i,f}\left(t\right)$ and standard variance $\sigma_{i,f}\left(t\right)$ of the time series CSI samples of the *f*-th subcarrier in the *i*-th transceiver antenna pair at time *t* can be calculated under the current window as follows:

(8) $$\mu_{i,f}\left(t\right)=\frac{1}{W}\sum_{p=t-3}^{t+3}x_{i,f}\left(p\right),$$

(9) $$\sigma_{i,f}\left(t\right)=\sqrt{\frac{1}{W}\sum_{p=t-3}^{t+3}{\left(x_{i,f}\left(p\right)-\mu_{i,f}\left(t\right)\right)}^{2}},$$

where $x_{i,f}\left(t\right)$ is the CSI amplitude or phase of the *f*-th subcarrier in the *i*-th transceiver antenna pair at time *t* and *W* is the time series window size. Based on the 3σ criterion, if $x_{i,f}\left(t\right)\notin \left[\mu_{i,f}\left(t\right)-3\sigma_{i,f}\left(t\right),\mu_{i,f}\left(t\right)+3\sigma_{i,f}\left(t\right)\right]$, then the current data are regarded as abnormal. Any such abnormal data are ticked and replaced by $\mu_{i,f}\left(t\right)$.

#### 3.3.3. Denoising

For background noise, a low-pass filter (i.e., Butterworth filter) is theoretically able to cancel high-frequency noise. However, conventional low-pass filters are not effective for removing burst and impulse noise, and a strict low-pass filter can result in the loss of useful signal [26]. Instead, we use a threshold-based denoising method based on wavelet decomposition [64]. This method can effectively protect spikes abrupt signals in the expected signals, remove burst and impulse noise, and suppress interference from high-frequency noise. Wavelet decomposition decomposes the CSI into two terms, the approximation coefficients and the detail coefficients; the former describe the shape of the CSI waveform, while the latter capture the noise and fine details of the CSI. In the wavelet domain, the effective CSI corresponds to large coefficients, while the noise corresponds to small coefficients and satisfies a Gaussian distribution. Thus, the threshold is predetermined such that the coefficients in a certain interval of the wavelet domain are set to zero and the high-frequency noise is suppressed.

We use the WAVEDEC function provided by the MATLAB wavelet toolbox to perform the signal decomposition. In this paper, we use the db4 wavelet function to decompose the CSI into three layers in order to obtain three detail coefficient vectors and one approximate coefficient vector. Meanwhile, the threshold is set as

(10) $$thr=\sqrt{2\times \log\left(m_{l}\right)},$$

where $m_{l}$ is the length of the CSI data samples and thr is the corresponding threshold value. The detail coefficients and approximate coefficients are processed by the hard threshold function, written as follows:

(11) $$w_{thr}=\left\{\begin{array}{ccc} w & \; & |w|\geq thr\\ 0 & \; & |w|<thr \end{array}\right.$$

where *w* represents the original wavelet coefficients and $w_{thr}$ the wavelet coefficients after hard threshold function processing. The wavelet coefficients after hard threshold function processing are then recombined and reconstructed to obtain the noise-reduced CSI.

**Figure 3:**
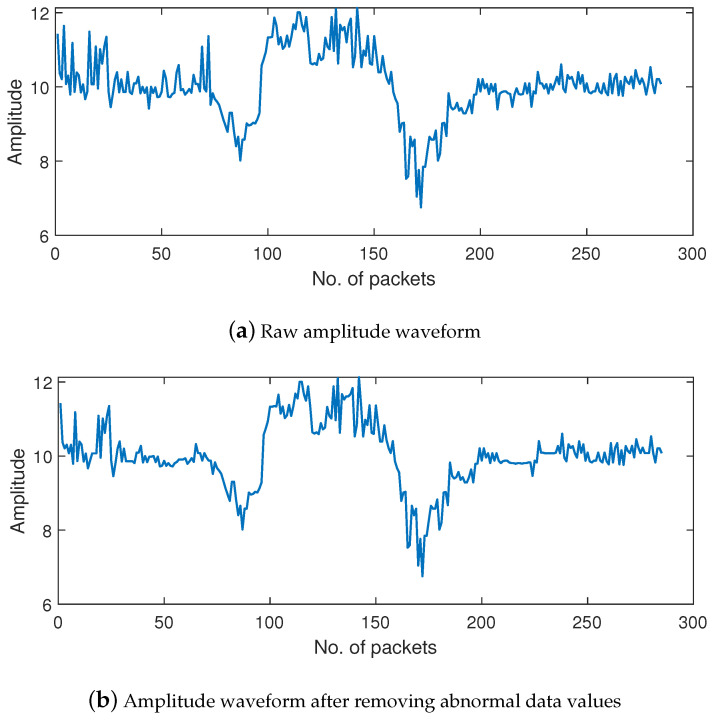

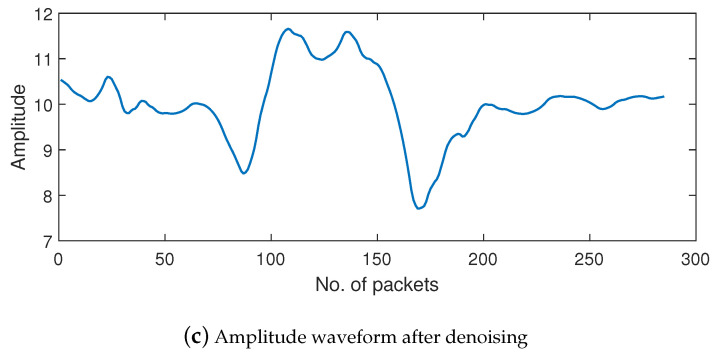
Amplitude waveform with “push and pull” gesture.

Figure 3 presents the CSI amplitude $A_{1,1}\left(t\right)$ before and after data processing. Figure 3a shows the original amplitude waveform obtained from the original measurement, containing both high-frequency noise and abnormal data. The waveform after replacing the abnormal data according to the 3σ criterion is presented in Figure 3b, and the waveform after removing the high-frequency noise by wavelet decomposition is shown in Figure 3c. It can be observed that the waveform of the processed CSI is smoother than that of the original CSI.

### 3.4. Feature Extraction and Selection

On the basis of the processed CSI, we chose the following six typical features in the time and frequency domains for both the CSI amplitude and phase: mean, variance, RMS, IQR, EE, and PSE. In the time domain, the mean and variance describe the average and uncertain trends of the CSI, with the latter also reflecting the degree of dispersion among individuals within the dataset. The RMS (known as the effective value) unveils the validity of the CSI. The IQR describes the state of the distribution of the data as a whole. In addition, two kind of frequency domain features are taken into account. The EE represents the energy distribution in different frequency bands of CSI data, while the PSE can reflect the spectral structure of the CSI.

Similar to [20], empirical mode decomposition (EMD) is employed for decomposition into several intrinsic mode functions (IMFs). The first eight IMFs, which contain the most dominant information, are selected and arranged as $c_{1}\left(t\right)$, $c_{2}\left(t\right)$, $c_{3}\left(t\right)$, $c_{4}\left(t\right)$, $c_{5}\left(t\right)$, $c_{6}\left(t\right)$, $c_{7}\left(t\right)$, $c_{8}\left(t\right)$ according to the frequency components, from highest to lowest. To this end, the EE is provided by

(12) $$EE=-\sum_{i=1}^{8}p_{i}\log p_{i},$$

where $p_{i}=\frac{E_{i}}{E}$ is the energy percentage, $E_{i}$ is the energy of $c_{i}\left(t\right)$, and $E=\sum_{i=1}^{8}E_{i}$ is the total energy.

Similar to [21], the discrete Fourier transform $X(\omega_{t})$ is obtained by performing the FFT on $A_{i,f}\left(t\right)$ or ${\tilde{\varphi }}_{i,f}\left(t\right)$, where $\omega_{t}$ is the frequency point at time *t*. Then, the power spectral density (PSD) is calculated as

(13) $$\hat{P}\left(\omega_{t}\right)=\frac{1}{N^{\prime }}{\left|X\left(\omega_{t}\right)\right|}^{2},$$

where $N^{\prime }$ is the number of frequency points of the FFT. The PSD distribution function is derived by normalizing $\hat{P}\left(\omega_{t}\right)$, i.e.,

(14) $$p_{t}=\frac{\hat{P}\left(\omega_{t}\right)}{\sum_{t}\hat{P}\left(\omega_{t}\right)}.$$

Then, the PSE is provided by

(15) $$PSE=-\sum_{t}p_{t}\log p_{t}.$$

To this end, we obtain 540 features for the CSI amplitude and 540 features for the CSI phase. Because noise and interference negatively affect the accuracy of gesture recognition, it may be the case that only part of the subcarriers can be chosen and used for gesture recognition. Thus, selecting effective features while eliminating redundant and irrelevant features, is an essential and significant step in improving recognition accuracy. In the feature selection phase, we use the improved Fisher score [65] to select the features. The Fisher score is a filter-based supervised feature selection method [66] which aims to find the most effective feature subspaces. In the traditional scheme, the score is calculated based on the ratio of the inter-class scatter $S_{b}\left(f_{i}\right)$ of the *i*-th feature $f_{i}$ and intra-class scatter $S_{t}^{\left(k\right)}\left(f_{i}\right)$ of the *k*-th class of gesture samples under the *i*-th feature $f_{i}$, which are provided by

(16) $$S_{b}\left(f_{i}\right)=\sum_{k=1}^{c}n_{k}{\left(\mu_{i}^{\left(k\right)}-\mu_{i}\right)}^{2},$$

(17) $$S_{t}^{\left(k\right)}\left(f_{i}\right)=\sum_{j=1}^{n_{k}}{\left(x_{i,j}^{\left(k\right)}-\mu_{i}^{\left(k\right)}\right)}^{2}=n_{k}\cdot {\left(\delta_{i}^{\left(k\right)}\right)}^{2},$$

(18) $$FS\left(f_{i}\right)=\frac{S_{b}\left(f_{i}\right)}{\sum_{k=1}^{c}S_{t}^{\left(k\right)}\left(f_{i}\right)}=\frac{\sum_{k=1}^{c}n_{k}{\left(\mu_{i}^{\left(k\right)}-\mu_{i}\right)}^{2}}{\sum_{k=1}^{c}n_{k}{\left(\delta_{i}^{\left(k\right)}\right)}^{2}},$$

where $n_{k}$ is the number of instances of the *k*-th class of gesture samples, *c* is the number of gesture types, $\mu_{i}^{\left(k\right)}$ is the mean of the *k*-th class of samples under the *i*-th feature $f_{i}$, $\mu_{i}$ is the mean of all samples under the *i*-th feature $f_{i}$, ${\left(\delta_{i}^{\left(k\right)}\right)}^{2}$ is the variance of the *k*-th class of samples under the *i*-th feature $f_{i}$, $x_{i,j}^{\left(k\right)}$ is the value of the *j*-th sample in the *k*-th class of samples taken under the *i*-th feature $f_{i}$, and $FS(f_{i})$ is the Fisher score of the whole feature dataset at the *i*-th feature $f_{i}$. An inter-class scatter $S_{b}\left(f_{i}\right)$ that is larger under the *i*-th feature $f_{i}$ indicates higher inter-class discrimination, while a smaller sum of the intra-class scatter $\sum_{k=1}^{c}S_{t}^{\left(k\right)}\left(f_{i}\right)$ of the classes *c* indicates a smaller intra-class error. Therefore, the larger the Fisher score under the *i*-th feature $f_{i}$, the stronger the discrimination of that feature for gestures. However, the traditional Fisher score has an obvious drawback in that the inter-class scatter $S_{b}\left(f_{i}\right)$ is obtained by subtracting the sample mean of each gesture type under the *i*-th feature from the total sample mean under the *i*-th feature, then squaring, and finally summing. This may not be reasonable; for example, when the difference between the sample mean of a gesture type under the *i*-th feature and the total sample mean under the *i*-th feature remains the same, the gesture type takes the same value for the *i*-th feature as the other gesture types take for the *i*-th feature, which results in both the inter-class scatter and the Fisher score remaining unchanged. In other words, the traditional Fisher score cannot reflect inter-class differences. Therefore, a correction is needed to account for the inter-class scatter, which is denoted by $NewS_{b}\left(f_{i}\right)$ as follows:

(19) $$o_{{kk}^{\prime }}^{\left(i\right)}=\frac{n_{k}+n_{k^{\prime }}-n_{{kk}^{\prime }}^{\left(i\right)}}{n_{c}},$$

(20) $$NewS_{b}\left(f_{i}\right)=\sum_{1\leq k\leq k^{\prime }\leq c}o_{{kk}^{\prime }}^{\left(i\right)}{\left(\mu_{i}^{\left(k\right)}-\mu_{i}^{\left(k^{\prime }\right)}\right)}^{2},$$

where $n_{c}=\sum_{k=1}^{c}n_{k}$ is the number of instances of all gesture samples, $\mu_{i}^{\left(k\right)}$ and $\mu_{i}^{\left(k^{\prime }\right)}$ are the means of the samples of the *k*-th and $k^{\prime }$-th classes under the *i*-th feature, respectively, $n_{k}$ and $n_{k^{\prime }}$ are the number of gesture samples of the *k*-th and $k^{\prime }$-th classes, respectively, $n_{kk^{\prime }}^{\left(i\right)}$ is the number of *k*-th and $k^{\prime }$-th classes of gesture samples that takes the same value under the *i*-th feature, and $o_{kk^{\prime }}^{\left(i\right)}$ is the cross-coefficient under the *i*-th feature. Therefore, the improved Fisher score is written as follows:

(21) $$NewFS\left(f_{i}\right)=\frac{\sum_{1\leq k\leq k^{\prime }\leq c}o_{kk^{\prime }}^{\left(i\right)}{\left(\mu_{i}^{\left(k\right)}-\mu_{i}^{\left(k^{\prime }\right)}\right)}^{2}}{\sum_{k=1}^{c}n_{k}{\left(\delta_{i}^{\left(k\right)}\right)}^{2}}$$

where $NewFS(f_{i})$ is the improved Fisher score, for which the intra-class scatter is calculated in the same way and the inter-class scatter is modified to be the sample mean of each gesture type under the *i*-th feature minus the sample mean of all other gesture type under the *i*-th feature. This improvement allows the score to better reflect inter-class differences. Similarly, a larger inter-class scatter $NewFS(f_{i})$ under the *i*-th feature $f_{i}$ and smaller sum of the intra-class scatter of the classes *c* indicates stronger discrimination of that feature for gestures.

**Figure 4:**
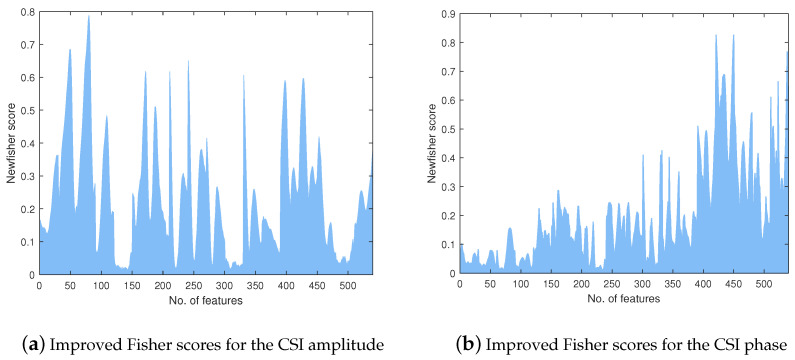
Improved Fisher scores of 540 features over all subcarriers.

Figure 4 presents the improved Fisher scores of the 540 features extracted from all subcarriers. Every 30 improved Fisher scores in the figure correspond to one feature, e.g., 1–30 are the means of the first antenna pair, 31–60 are the variances of the first antenna pair, etc. It can be seen that not all subcarriers have high scores, which implies that the features with low scores are less sensitive to gestural movements compared to the features of higher scores. It can also be seen that the EE of low scores (121–150, 301–330, 481–510) cannot distinguish each gesture very well. To reduce those features that cannot effectively distinguish gestures, we empirically chose 0.12 as the threshold. Features with scores below this threshold were removed.

In the next step, PCA is used to eliminate the redundant features, further reducing the computational cost of the subsequent training procedure. The PCA method converts the original feature vector into a set of linearly uncorrelated principal components and eliminates the redundant components. The details of PCA can be summarized as follows:

- **Step 1: Calculate the covariance matrix.** The covariance of the features selected based on the improved Fisher score is calculated and a covariance matrix with dimension $n_{s}\times n_{s}$ is constructed, where $n_{s}$ is the number of selected features.
- **Step 2: Calculate the eigenvalues and eigenvectors.** The eigenvalues *e* and eigenvectors *q* of the covariance matrix are calculated, then the contribution scores are obtained based on the eigenvalues. The contribution scores are ranked, and all of the eigenvalues and eigenvectors with contribution scores up to 0.95 are selected.
- **Step 3: Calculate the principal components.** Using the eigenvalues and eigenvectors selected in Step 2, the linearly uncorrelated principal components can be obtained by decomposing them with F×q (where *F* is the dataset of features after feature selection) for use as the training set in the next step of model training.

### 3.5. Training and Gesture Recognition

The extracted and selected features can now be used in machine learning to train a gesture recognition classification model. According to the CSI Fresnel zone theorem, the CSI amplitude and phase are a pair of complementary signals [19] that have different sensitivities to gestures. In this paper, we jointly exploit the features of the CSI amplitude and phase to obtain a gesture recognition classification model. In addition, we propose an SVM-Stacking algorithm based on the stacking algorithm concept, wherein the averaged probability estimation vectors of both models for CSI amplitude and phase are exploited as the input to train a meta-model for classification fusion estimation.

SVM is a supervised based machine learning method that solves nonlinear classification problems using kernel functions [67]. Hence, it can be applied to the gesture recognition classification problem in this paper to train a phase model and amplitude model, then average the probability estimation vectors of the two models. The traditional SVM can only handle binary classification simultaneously, and requires the dataset to be linearly separable. As the gesture recognition classification in this paper is a multi-classification problem, some modifications are needed. Multi-classification with SVM can be achieved by using a one-versus-one (OVO) strategy. First, the dataset is assigned c classes; then, a classifier is trained for each two of the c classes, yielding a total of $\frac{c\cdot \left(c-1\right)}{2}$ binary classifiers. Finally, when classifying a sample of an unknown class, it is put into $\frac{c\cdot \left(c-1\right)}{2}$ binary classifiers, and the class with the most votes is assigned as the class of the unknown sample.

With the development of classification algorithms such as SVM, better classification results can be realized. However, these algorithms can easily fall into overfitting situations. Ensemble learning can effectively alleviate the overfitting problem for traditional classification algorithms. As a special ensemble learning algorithm, the stacking algorithm combines multiple individual learners to realize higher classification accuracy. The individual learner and the learner used for combination are called the primary and secondary learners, respectively. In this paper, there are two kind of primary learners, namely, a phase-based training model and an amplitude based training model, and the secondary learner is used to train a meta-model by taking the averaged probability estimation vectors of the two models as the input.

**Figure 5:**
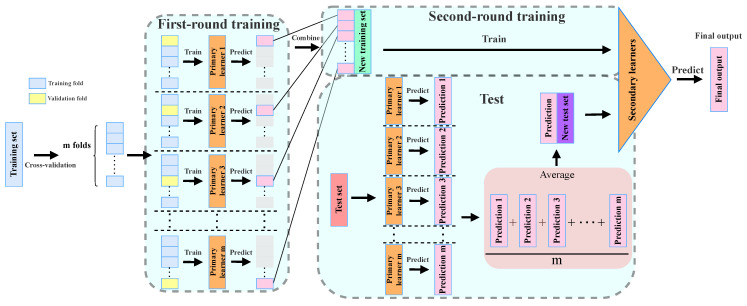
Structure diagram of a generic stacking algorithm.

The structure of the generic stacking algorithm is presented in Figure 5, with the details summarized below:

- First, as there are m primary learners, the training dataset is randomly divided into m folds.
- Second, in the first round of training, for the *i*-th ($i\in \left\{1,2,3,\cdots ,m\right\}$) primary learner, we select the corresponding *i*-th fold as the validation fold and the remaining m-1 folds as the training folds. Subsequently, we train the *i*-th primary learner, resulting in a total of m primary learners.
- Third, each primary learner makes primary predictions on the corresponding validation fold. These primary predictions are reconstructed to form a new training set for the secondary learner, which is then obtained through a second round of training.
- Fourth, the test set is fed to the m primary learners, resulting in m sets of primary predictions. These primary prediction sets are then averaged to form a new test set for the secondary learner.
- Finally, the secondary learner is used to make predictions on the new test set and obtain the final output.

Stacking is easily scalable and can be used to build algorithmic models for different tasks. Therefore, in this paper we apply it to combine the complementary advantages of the phase and amplitude, which can efficiently improve the training of gesture classification models while effectively alleviating the overfitting problem. The details of our SVM-Stacking algorithm are described in Algorithm 1, which consists of two main stages. In the first stage, we use the training set of the CSI phase data samples to train the primary phase learner and use the amplitude training set to train the primary amplitude learner. In the second stage, we use the two primary learners obtained in the first stage to train the secondary learner. We put the validation set of the phase into the primary phase learner to derive the phase probability estimation vectors and put the amplitude validation set into the primary amplitude learner to obtain the corresponding probability estimation vectors. Based on the complementarity of the CSI phase and amplitude, we average these two probability estimation vectors and combine the corresponding class labels as a new training set, which is used to train the secondary learners. Finally, the SVM-Stacking classification model is generated.
**Algorithm 1:** SVM-Stacking algorithm for gesture classification
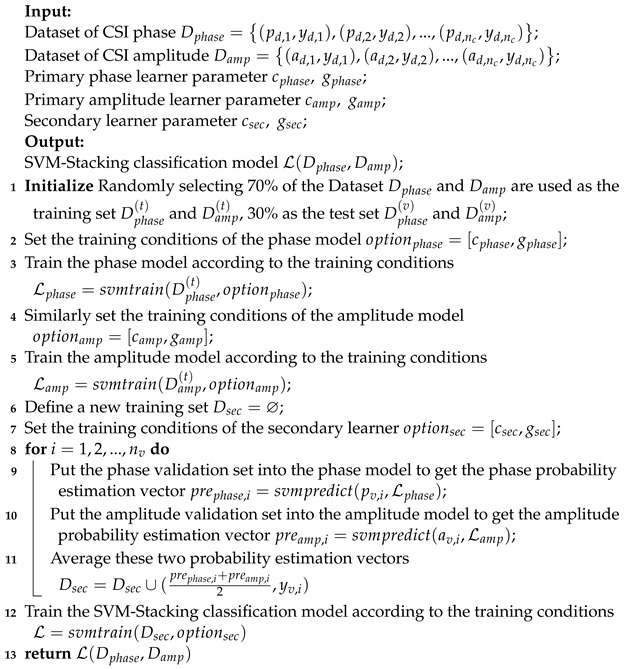


## 4. Performance Evaluation

In this section, we describe the experiments conducted to evaluate the performance achieved by the proposed gesture recognition system.

### 4.1. Experimental Setting

**Figure 6:**
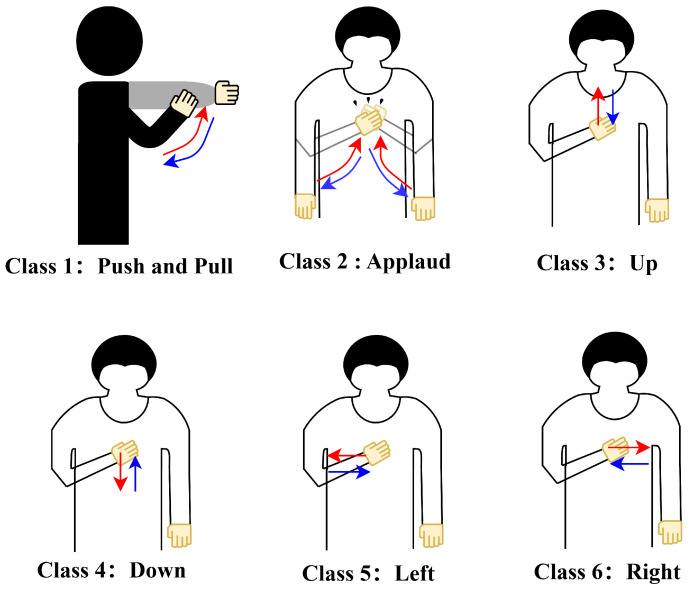
Sketch of six gestures, where the start and end directions are the directions of the red and blue lines, respectively.

In the experiments, an AP with an omnidirectional antenna was used as the transmitter and a minicomputer equipped with an Intel 5300 NIC was deployed as the receiver. In addition, the operating system of minicomputer was Ubuntu 14.04. We used the linux-802.11n-csitool to modify the driver to collect the CSI sample data in the NIC. While the experiment was in progress, the transmitter continuously sent data to the receiver at a rate of 100 CSI samples per second. In the collected samples, the CSI is a two-dimensional complex matrix with size 3×30, where 3 stands for the three receiving antennas and 30 represents the thirty subcarriers. The experiment was conducted in a square room with dimensions of 7.7 × 7 m^2^, and the AP and minicomputer were both deployed at a height of 1 m. During the experiment, there were five volunteers, each of whom stayed in the testing area while completing a gesture within 3 s. Figure 6 shows six typical hand gestures. In order to achieve a higher generalization capability, each volunteer was selected randomly, meaning that we did not intentionally focus on characteristics such as height, weight, or body contour.

### 4.2. Recognition Accuracy Performance

For the SVM algorithm, we used the RBF kernel function and employed a grid search approach to optimize both the reward and penalty parameters for model tuning. We used a PC equipped with an Amd Ryzen 7 4800H central processing unit (CPU) with Radeon Graphics during data processing and training. At the same time, we used cross-validation to train and validate the model. Ultimately, to improve the robustness of the model, we did not impose a strict limit on the hand speed of volunteers, only requiring that gestures be completed within 3 s. Moreover, we have shared all our source codes and datasets at https://github.com/hn86441283/Device-free-Wireless-Sensing-for-Gesture-Recognition-based-on-Complementary-CSI-Amplitude-and-Phase/tree/master to facilitate further validation and optimization.

**Figure 7:**
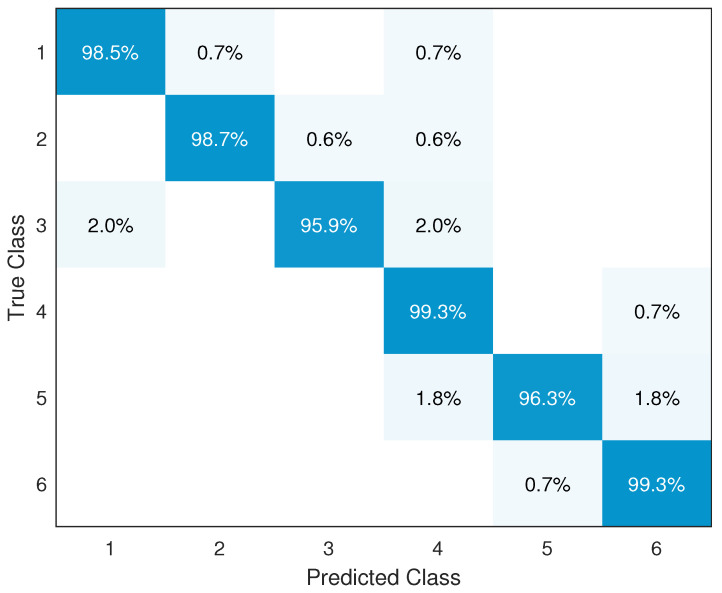
Confusion matrix of classification results.

In the proposed gesture recognition system, six features are extracted: mean, variance, RMS, IQR, EE, and PSE. After feature selection is performed, these features are used in the the SVM-Stacking classifier. On average, the gesture recognition accuracy approaches 98.3%. The confusion matrix of the classification results for the six gestures is shown in Figure 7. Our system produces stable performance on different gestures, with recognition accuracy ranging from 95.9% to 99.3% with a standard deviation of 0.0151129.

**Figure 8:**
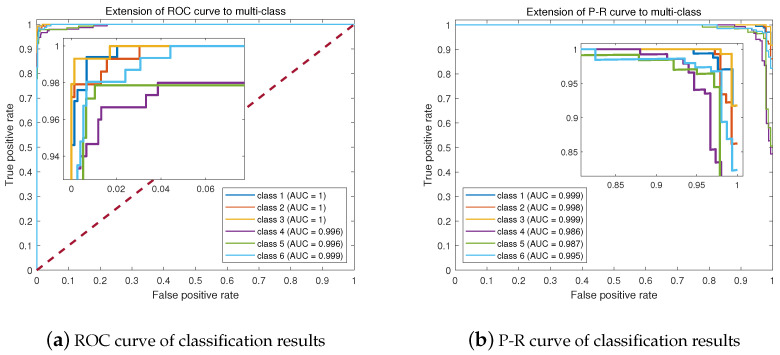
ROC and P-R curves of the classification results.

The precision and recall of six gestures are evaluated in Table 2. The lowest recall for the six gestures is higher than 95%, and the lowest precision is higher than 94%. The average precision and recall for the six gestures reaches 98.3%, demonstrating the good accuracy of our system in recognizing various gestures. Figure 8a,b respectively present the Receiver Operating Characteristic (ROC) curve and the Precision–Recall (P-R) curve of the SVM-Stacking classification for the six classes. It can be seen that the values of the area under the curve (AUC) for the six classes all converge to 1 or are equal to 1. The closer the AUC value is to 1, the better the model’s performance; thus, these results indicate that the classifier is able to accurately recognize the gestures with minimal error. Meanwhile, the P-R curve characterizes the relationship between precision and recall; similar to the ROC curve, a larger area under the curve indicates better performance on the part of the classifier.

### 4.3. Impact of Feature Selection and Classification Methods

**Figure 9:**
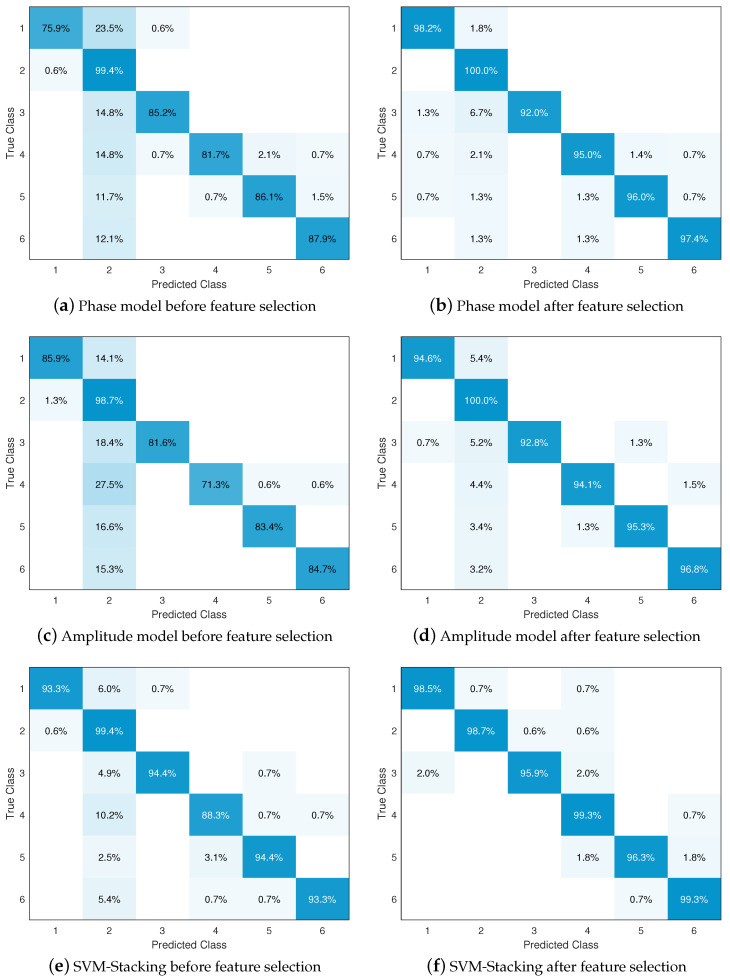
Confusion matrix of the classification results.

In this part, we evaluate the performance of our system with and without feature selection and stacking in order to evaluate their effectiveness. The accuracy comparisons of the phase classification model, amplitude classification model, and SVM-Stacking classification model are shown in Table 3, and the confusion matrix of the results is shown in Figure 9. It can be seen that fewer instances are predicted incorrectly after the model undergoes feature selection compared to no feature selection; the overall accuracy is improved by an average of 8.7%, while the computation time (including grid optimization) decreases by 89.57%. In particular, the number of iterations of the model and the total number of support vectors are both reduced, implying a decrease in the complexity of the model. The feature selection operation calculates the contribution score of each feature in order to distinguish the gesture, then finally selects only those features that can effectively distinguish the gesture, which helps to reduce the useless and redundant features, thereby improving accuracy and reducing computational cost. In addition, the stacking algorithm significantly outperforms the separate phase and amplitude models, with an average increase of 5.6% in overall accuracy. The stacking algorithm is used train a meta-model based on the complementarity of the phase and amplitude by averaging the probability estimation vectors of the phase and amplitude models. An interesting phenomenon is that although the stacking algorithm is used to integrate the phase and amplitude models, the computation time (including grid optimization) is not a superposition of the time of the two models, but is instead a compromise between the times of the two models. Thus, there is only a slight improvement in the number of iterations and total number of expenditure vectors, as when integrating two models the training set of a single model continues to be split into a training set and a test set. The experimental results show that the proposed gesture recognition system using feature selection and stacking algorithm can accurately estimate the class of gestures from the feature data.

Here, we compare the performance of the proposed feature selection algorithm with those of two classical feature selection algorithms: Lasso [68] and ReliefF [69]. Lasso is an embedded method, while ReliefF is a wrapper methods. In Lasso, we performed ten-fold cross-validation in the range $\left[2^{-5},2^{0}\right]$ and choose the lambda corresponding to the minimum cross-validated mean squared error for feature selection. In ReliefF, we chose to use 100 similar samples as nearest neighbors. Because the feature selection module is only a part of our system, we show the performance difference between the different feature selection algorithms by comparing the performance of the system after using the algorithms. The performance comparison results are shown in Table 4, which reveals that the improved Fisher score slightly outperforms the ReliefF and Lasso algorithms in terms of both overall accuracy and computational efficiency. This is to the improved Fisher score paying more attention to the differences between categories. Furthermore, integrating the improved Fisher score with the PCA method greatly improves the computational efficiency, while the recognition accuracy remain basically unchanged. This is because the PCA method converts the original feature vectors into a set of linearly uncorrelated principal components while eliminating the redundant components.

### 4.4. Performance Comparison

**Figure 10:**
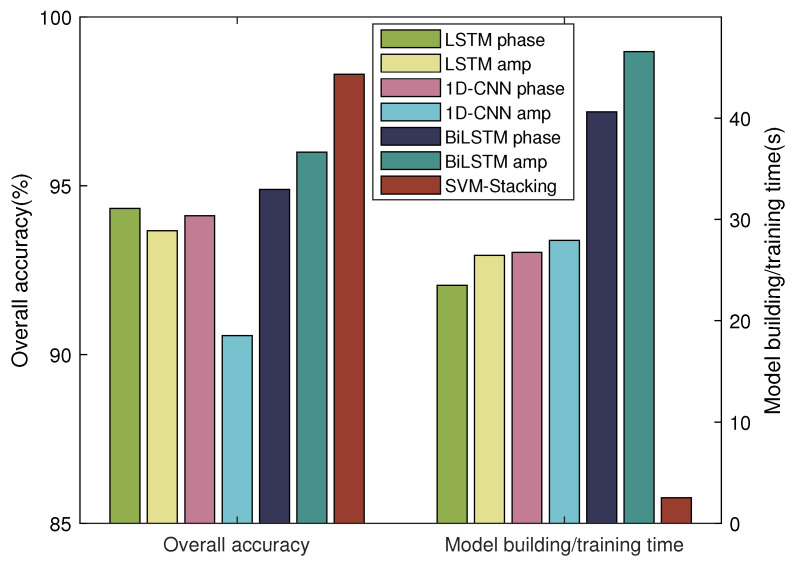
Performance comparison of different classification algorithms.

Next, we compare the performance of the proposed classification algorithm with those of other existing algorithms, including LSTM, 1D-CNN, and BiLSTM. The LSTM network contained one LSTM hidden layer and 128 hidden units. In the 1D-CNN model, two Conv1D layers with 32 filters of size 5 were deployed and ReLU was used as the activation function after each Conv1D layer. For BiLSTM, we used one BiLSTM layer with 200 hidden units and used ReLU as the activation function afterwards. Because these classification algorithms cannot utilize the CSI phase and amplitude jointly, either phase features or amplitude features were used. The results of the performance comparison are presented in Figure 10. It can be seen that the SVM-Stacking algorithm performs best due to joint utilization of the complementary CSI phase and amplitude. Meanwhile, because the neural networks require extensive computational resources for training the classification models, SVM-Stacking shows great benefits in terms of reduced execution time.

**Figure 11:**
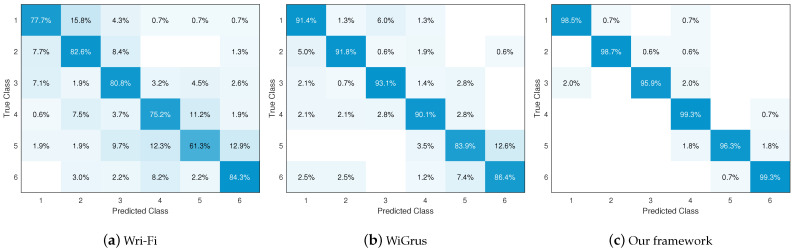
Confusion matrix results of the three schemes.

**Figure 12:**
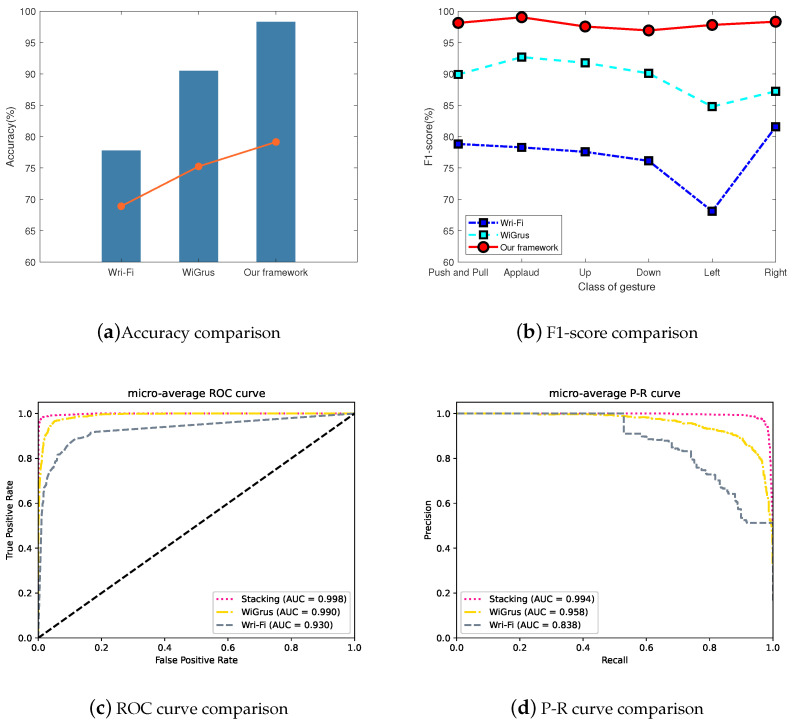
Comparison of the three scenes.

Lastly, we compare our proposed system with two other schemes: WiGrus [18], which uses a two-stage random forest algorithm for classification of gestures, and Wri-Fi [13], which uses a combination of a Gaussian mixture model and a hidden Markov model for the classification of air-handwritten alphabets. We applied both schemes to the dataset obtained from our experiments. The confusion matrix results are shown in Figure 11, from which it can be observed that our proposed system has the least instances of estimation error. The overall classification results and comparison are shown in Figure 12a, where it can be seen that our proposed system reaches an accuracy of 98.3%, compared to 90.5% and 77.8% for the other schemes. The F1-scores of the three schemes are shown in Figure 12b, where it can be seen that our proposed system exceeds 97% with minimal fluctuations. In addition, the micro-average ROC and P-R curves of the classification results yielded by the three schemes are shown in Figure 12c,d, respectively; it can be seen that the results of our proposed system are optimal for both curves. All of these results validate the effectiveness of our proposed gesture recognition system.

## 5. Discussion and Conclusions

In this section, we discuss the research limitations and challenges around this topic based on the above results, then conclude with the main findings of this paper.

### 5.1. Discussion

The experimental results presented above validate the effectiveness of the proposed HGR system based on exploiting the complementary CSI amplitude and phase. However, there are some research limitations and challenges around both our work and the research topic as a whole which need to be addressed in the future. These are listed below:

1. Robustness and generality issues for real-world applications. The research work in this paper, as well as most of the existing research work, collects CSI data on different gestures in a relatively ideal environment, and subsequent processing steps are conducted based on machine learning or deep learning. However, the practical environment usually changes over time, for instance, changes in the external scene of the gesture action or the speed and angle of human movements, and the CSI is highly sensitive to the above factors. Thus, how to improve robustness in practical application scenarios remains one of most significant research challenges in the field of wireless sensing technology. In general, large-scale and high-quality datasets containing the CSI of samples taken from diverse and dynamic environments are necessary in order to address the robustness issue.
2. Practical wireless sensing applications within the edge computing paradigm. In order to truly apply WiFi-based gesture recognition technology to real life, it is necessary to consider the load of the central server along with data transmission delay and other issues. Recent developments in edge computing technology allow part of the data processing and analysis tasks of edge devices to be offloaded to the edge server, which can reduce the load on the central server and reduce data transmission delays [70]. Therefore, integrating wireless sensing technology into the edge computing paradigm represents a critical way to deploy WiFi-based gesture recognition technology into daily life.
3. Issues with recognizing multiple gestures in complex environments. When multiple peoples perform gestures in a complex environment, channel variations and interference caused by their bodies may be superimposed, leading to complex signal aliasing. In addition, multiple gestures made by multiple peoples may lead to cross-interference, where the gesture of one individual may affect the signal of another. This makes it challenging to extract effective gesture features, leading to a decrease in gesture recognition performance.

### 5.2. Conclusions

This paper has developed a wireless sensing-based human gesture recognition system which jointly exploits the complementary CSI amplitude and phase to improve the recognition accuracy. Meanwhile, a feature selection algorithm based on an improved Fisher score is utilized to construct an effective feature subspace for distinguishing different gestures, then an SVM-Stacking algorithm is proposed for gesture classification. Finally, our experiments validate the robustness of the proposed system, reaching an average accuracy of 98.3%, which outperforms other recent schemes.

In the future, our work will seek to use techniques from generative adversarial networks (GAN) or transfer learning to generate samples of new gestures by learning and utilizing features and representations of existing gestures to synthesize and train samples of gestures outside the dataset. This will allow the model to learn a wider range of gesture patterns, thereby improving its recognition and understanding of unknown gestures. Concerning the issue of limited coverage, complex ultra-dense network scenarios with multiple access points (APs) can enlarge the coverage of wireless sensing applications and improve their practicability. Furthermore, in this work we have considered a sensing scenario consisting of a single transmitter–receiver pair. In the future, we will continue this work by considering a more complex scenario and the design of robust device-free wireless sensing approaches. These research efforts can enhance the practical deployment of wireless sensing technologies and improve the quality of daily life.

## Figures and Tables

**Table 1:** Definitions of symbols and notations.

Symbol	Definition	Symbol	Definition
H(t)	The CSI at time *t*	$H_{i,f}\left(t\right)$	The CSI of the *i*-th transceiver antenna pair in the *f*-th subcarrier at time *t*
$X_{i,f}\left(t\right),Y_{i,f}\left(t\right)$	The transmitted and received frequency signals of the *i*-th transceiver antenna pair in the *f*-th subcarrier at time *t*	$A_{i,f}\left(t\right),\;\varphi_{i,f}\left(t\right)$	The amplitude and phase of $H_{i,f}\left(t\right)$
${\dot{\varphi }}_{i,f}\left(t\right)$	The measured CSI phase of the *i*-th transceiver antenna pair in the *f*-th subcarrier at time *t*	$k_{f}$	The subcarrier index varying from −28 to 28 in IEEE 802.11n
*N*	The number of (FFT) window points	δ(t)	The time offset between the transmitter and receiver at time *t*
β(t)	The unknown phase offset at time *t*	Z(t)	The noise introduced by the measurement procedure at time *t*
$a_{i}(t),\;b_{i}\left(t\right)$	The two variables introduced to realize the linear transformation of the *i*-th transceiver antenna pair at time *t*	*n*	The total number of subcarriers
${\tilde{\varphi }}_{i,f}\left(t\right)$	The calibrated CSI phase of the *i*-th transceiver antenna pair in the *f*-th subcarrier at time *t*	$\mu_{i,f}\left(t\right),\sigma_{i,f}\left(t\right)$	The mean and variance of the *i*-th transceiver antenna pair in the *f*-th subcarrier at time *t* under the window
$x_{i,f}\left(t\right)$	The CSI phase or CSI amplitude of the *i*-th transceiver antenna pair in the *f*-th subcarrier at time *t*	*W*	The time series window size
$m_{l}$	The length of CSI data samples	thr	The threshold value
$w,w_{thr}$	The original wavelet coefficients and processed wavelet coefficients	$c_{i}\left(t\right)$	The *i*-th IMFs
*E*	The total energy of all IMFs	$E_{i},\;p_{i}$	The energy and energy percentage of $c_{i}\left(t\right)$
$X(\omega_{t})$	The FFT on $A_{i,f}\left(t\right)$ or ${\tilde{\varphi }}_{i,f}\left(t\right)$	$\omega_{t}$	The frequency point at time *t*
$N^{\prime }$	The number of frequency points of the FFT	$\hat{P}\left(\omega_{t}\right)$	The PSD
$p_{t}$	The PSD distribution function	$S_{b}\left(f_{i}\right)$	The inter-class scatter of *i*-th feature $f_{i}$
$S_{t}^{\left(k\right)}\left(f_{i}\right)$	The intra-class scatter of *k*-th class of gesture samples under *i*-th feature $f_{i}$	$FS(f_{i})$	The Fisher score of the whole feature dataset at the *i*-th feature $f_{i}$
$n_{c}$	The number of instances of all gesture samples	*c*	The number of gesture types
$\mu_{i}$	The mean of all samples under the *i*-th feature $f_{i}$	${\left(\delta_{i}^{\left(k\right)}\right)}^{2}$	The variance of the *k*-th class of samples under the *i*-th feature $f_{i}$
$x_{i,j}^{\left(k\right)}$	The value of the *j*-th sample in the *k*-th class of samples taken under the *i*-th feature $f_{i}$	$NewS_{b}\left(f_{i}\right)$	The inter-class scatter of *i*-th feature $f_{i}$ after correction
$\mu_{i}^{\left(k\right)},\;\mu_{i}^{\left(k^{\prime }\right)}$	The means of the samples of *k*-th and $k^{\prime }$-th class under the *i*-th feature	$n_{k},\;n_{k^{\prime }}$	The number of gesture samples of *k*-th and $k^{\prime }$-th class
$n_{{kk}^{\prime }}^{\left(i\right)}$	The number of *k*-th and $k^{\prime }$-th class of gesture samples that takes the same value under the *i*-th feature	$o_{{kk}^{\prime }}^{\left(i\right)}$	The cross coefficient under the *i*-th feature
$NewFS(f_{i})$	The improved Fisher score at the *i*-th feature $f_{i}$	*F*	The data set of features after feature selection
$n_{s}$	The number of selected features	e, q	The eigenvalues and eigenvectors of the covariance matrix
*m*	The number of folds in stacking algorithm	$D_{phase},\;D_{amp}$	Dataset of CSI phase and amplitude
$a_{d,i},\;y_{d,i}$	Amplitude feature vector and gesture label	$p_{d,i},\;y_{d,i}$	Phase feature vector and gesture label
$c_{phase},\;g_{phase}$	Primary phase learner parameter	$c_{amp},\;g_{amp}$	Primary amplitude learner parameter
$c_{sec},\;g_{sec}$	Secondary learner parameter	L	SVM-Stacking classification model
$D_{phase}^{\left(t\right)},\;D_{amp}^{\left(t\right)}$	Training dataset of CSI phase and amplitude	$D_{phase}^{\left(v\right)},\;D_{amp}^{\left(v\right)}$	Validation dataset of CSI phase and amplitude
$n_{v}$	The number of validation dataset	$L_{phase},\;L_{amp}$	The primary phase model and the primary amplitude model
$D_{sec}$	The secondary model training set	${pre}_{phase,i},\;{pre}_{amp,i}$	The phase model and amplitude model probability estimation vector

**Table 2:** Precision and recall of recognition for six gestures.

Value	Class of Gesture					
	Class1	Class2	Class3	Class4	Class5	Class6
**Recall**	98.5%	98.7%	95.9%	99.3%	96.3%	99.3%
**Precision**	97.3%	99.4%	99.3%	94.7%	99.4%	97.4%

**Table 3:** Comparison of the phase classification model, amplitude classification model, and SVM-Stacking classifier before and after feature selection.

Method		Accuracy							Computing Time	Phase Iter	Amplitude Iter	Meta Iter	Phase nSV	Amplitude nSV	Meta nSV
		Class1	Class2	Class3	Class4	Class5	Class6	Overall							
**Before feature** **selection**	Phase model	75.9%	99.4%	85.2%	81.7%	86.1%	87.9%	86.1%	405.06 s	1060	—	—	1818	—	—
	Amplitude model	85.9%	98.7%	81.6%	71.3%	83.4%	84.7%	84.1%	384.29 s	—	1092	—	—	2066	—
	SVM-Stacking classifier	93.3%	99.4%	94.4%	88.3%	94.4%	93.3%	94.0%	323.66 s	740	855	72	1351	1457	83
**After feature** **selection**	Phase model	98.2%	100%	92.0%	95.0%	96.0%	97.4%	96.4%	47.87 s	840	—	—	1434	—	—
	Amplitude model	94.6%	100%	92.8%	94.1%	95.3%	96.8%	95.6%	27.99 s	—	959	—	—	1500	—
	SVM-Stacking classifier	98.5%	98.7%	95.9%	99.3%	96.3%	99.3%	98.3%	39.44 s	614	675	43	1140	1144	65

**Table 4:** Performance comparison of different feature selection algorithms.

Method	Overall Accuracy	Computing Time	Phase Iter	Amplitude Iter	Meta Iter	Phase nSV	Amplitude nSV	Meta nSV
ReliefF	97.7%	245.36s	790	836	50	1230	1313	88
Lasso	96.8%	225.43s	753	790	31	1329	1447	59
NewFisher	98.1%	217.50s	697	739	48	1239	1243	80
NewFisher + PCA	98.3%	39.44s	614	675	43	1140	1144	65

## Data Availability

Not applicable.
